# Eleven-Year Distribution Pattern of Hepatitis C Virus in Southern Italy

**DOI:** 10.1155/2012/631095

**Published:** 2012-08-09

**Authors:** Nadia Marascio, Giovanni Matera, Angela Quirino, Aida Giancotti, Giorgio S. Barreca, Angelo G. Lamberti, Benedetto Caroleo, Maria Carla Liberto, Alfredo Focà

**Affiliations:** ^1^Department of Health Sciences, Institute of Microbiology, University “Magna Graecia” of Catanzaro, Viale Salvatore Venuta, Germaneto, 88100 Catanzaro, Italy; ^2^Department of Experimental and Clinical Medicine, Institute of Infectious Diseases, University “Magna Graecia” of Catanzaro 88100, Catanzaro, Italy

## Abstract

Analysis of the Hepatitis C Virus (HCV) genotype spread in a particular area has a crucial impact on public health. In this study, we update information on the distribution of HCV genotypes, by evaluating a hospital-based cohort of 2,153 chronic hepatitis C patients, collected prospectively among subjects attending University Hospital of Catanzaro, within an area of Southern Italy. We assessed the rates (%) of HCV genotypes during two consecutive periods, from 2001 to 2005 and from 2006 to 2011, according to age and gender. Considering overall observation time, subtype 1b was predominant followed by subtypes 2a/2c, genotype 3 and 4. Statistical evaluation of the age of HCV patients stratified by genotypes, revealed a slight but significant increase in the median age of 1b, 2a/2c and 3 HCV genotype-infected subjects, during the 2006–2011 period, whilst genotype 4 patients exhibited a decrease in the median age during the same period studied. Moreover genotype 4 increased between 2002 and 2003 as well as between 2010 and 2011. Due to the peculiar diagnostic/clinical/therapeutic features of HCV-4, our findings warrant a deeper investigation to better control infections caused by such genotype.

## 1. Introduction

Hepatitis C virus (HCV) is a major cause of acute and chronic liver disease worldwide. In as many as 85% of cases, acute HCV infection progresses to a chronic hepatitis. Infection by HCV constitutes an important health problem, and its spread reflects the socioeconomic standards and good sanitary regulations [1]. The distribution of HCV depends on a complex interaction between the social risks of the host and the molecular characteristics of the virus. HCV is characterized by a high degree of genetic heterogeneity; indeed, it is classified into three hierarchical layers based on a decreasing extent of different nucleotide variation: genotypes, subtypes, and quasispecies. HCV genotype distribution reflects the epidemiology of hepatitis C, and as such is related to particular routes of transmission.

 Subtype 1b is observed worldwide, whereas genotypes 1a and 3a are distributed in European and North American countries, genotype 2 in the Mediterranean region, Far East, and Western Africa. Genotype 4 is endemic in Middle East and Central Africa, genotype 5 in South Africa, genotype 6 in South East Asia [2], and genotype 7 was found in patients from the Democratic Republic of Congo [3]. In Europe, the most common subtype is 1b followed by subtype 2a/2c and genotype 3. Among European countries, the highest prevalence of HCV infection has been found in Italy. Such prevalence is ranging between 3 and 26%, and a progressive increase along with age has been observed, in particular in southern regions of Italy [4, 5]. Our group studied HCV genotype distribution in previously under-evaluated areas of South Italy [6]. Because of combination of several factors, such as improvement in health-care-related standards, eradication of transfusion-associated infections, and enhancement in immigration from endemic areas, possible changes in distribution of HCV genotypes over the last decade could be conceivable. Therefore, the aim of this study was to update the findings on the distribution of hepatitis C virus genotypes, by evaluating a hospital-based cohort of 2,153 chronic hepatitis C patients, collected prospectively among subjects attending University Hospital of Catanzaro, within an area of Southern Italy. 

## 2. Patients and Methods

During the 2001–2011 period, serum samples from 2,153 new consecutive HCV RNA positive patients, attending the University Hospital of Catanzaro, were collected. All patients enrolled in the present study were born in Italy and resident in Calabria. Among the enrolled subjects 1,148 (53.3%) of them were male and 1,005 (46.7%) were female; the mean age was 58.1 ± 15.2 (range 5–93 years). No patient was treated before sample collection. The enrollment distribution per each year during the study period is shown in Table 1. The patients added to the study each year are reported as total for each year at the bottom of each column of Table 1. Also these patients are new ones, who never have been enrolled in other years. Moreover, the two periods considered allowed us to more closely analyze the possible changes in the rate of the different HCV genotypes, as reported in the literature with some modifications [9–11]. Data on risk factors for HCV infection, when available, included blood transfusions, surgical procedures, drug addictions and sporadically acquired infections. For each patient an anonymous schedule was completed, including age and sex. Written informed consent was obtained from enrolled subjects or, where participants were children, from a parent or guardian. The study was approved by University “Magna Graecia” of Catanzaro Ethical Committee and Institutional Review Board. 

The Cobas AmpliPrep/Cobas TaqMan HCV test (Roche Diagnostics, Milan, Italy) has been used for extraction and detection of HCV RNA [7]. To search the viral RNA in samples collected before April 2006, the Cobas Amplicor HCV monitor test, version 2.0 (Roche Diagnostics, Milan, Italy) was used [8]. The amplified region of HCV genome was the 5′-UTR following the producer recommendations. PCR products were analyzed directly for genotyping by line probe assay (Versant HCV genotype 2.0 assay (LIPA) by Siemens, Healthcare Diagnostic Inc., Tarrytown, NY, USA, formerly Bayer Healthcare LLC, Tarrytown, NY, USA, formerly Innogenetics, Ghent, Belgium) based on reverse-hybridization, according to the manufacturer's protocol. Genotypes were classified according to Simmonds et al. [2]. Mann-Whitney test and the *χ* ^2^ analysis were used, where appropriate and performed using GraphPad Prism version 4.00 for Windows (GraphPad Software, San Diego, CA, USA, http://www.graphpad.com/). Differences were considered significant for *P* < 0.05.

## 3. Results

The distribution of HCV genotypes in 2,153 HCV new positive patients attending Catanzaro University Hospital and stratified by gender is shown in Table 2. Subtype 1b was the most prevalent (49.2%) followed by subtype 2a/2c (22.4%). There were no significant (*P* > 0.05 by *χ* ^2^) gender-related variation in the distribution of subtypes 1b and 2a/2c. Genotype 3 was the third most frequent (7.4%) and was significantly more common in male patients (83.1% versus 16.9% female, *P* < 0.05 by *χ* ^2^ test). Genotype 4 showed a rate of 6.2%, without a significant male versus female difference (58.2% male versus 41.8% female, *P* > 0.05 by *χ* ^2^ test) followed by subtype 1a and genotype 1 (4.2%), subtype 1a/1b (3.3%) and genotype 2 (3.0%). Three patients were infected by HCV genotype 5 (0.1%) and no patients by genotype 6.

**Figure 1:**
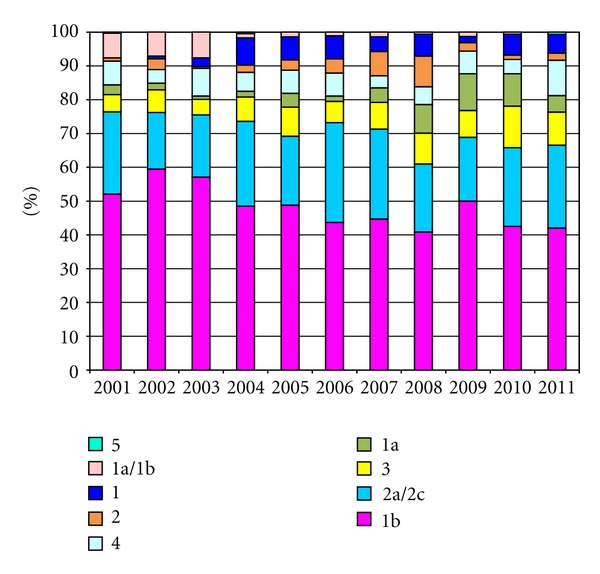
Dynamic distributions of HCV genotypes from January 2001 to December 2011. The Cobas AmpliPrep/Cobas TaqMan HCV test (Roche Diagnostics, Milan, Italy) has been used for extraction and detection of HCV RNA [7]. To search the viral RNA in samples collected before April 2006, the Cobas Amplicor HCV monitor test, version 2.0 (Roche Diagnostics, Milan, Italy) was used [8]. The amplified region of HCV genome was the 5′-UTR following the producer recommendations. PCR products were analyzed directly for genotyping by line probe assay (Versant HCV genotype 2.0 assay (LIPA) by Siemens, Healthcare Diagnostic Inc., Tarrytown, NY, USA, formerly Bayer Healthcare LLC, Tarrytown, NY, USA, formerly Innogenetics, Ghent, Belgium) based on reverse-hybridization, according to the manufacturer's protocol.

The dynamics of HCV genotype distribution during the 11-year period studied is reported in Figure 1. Also the total number of patients added per each year is reported in Table 1 at the bottom of each column. Subtype 1b decreased starting from 2001 (52.1%) and 2002 (59.5%) to 2011 (42.0%), with exception of 2009 rate, while subtype 2a/2c did not vary significantly with time (24.3% versus 24.5%). Genotype 3 showed a peak of 12.3% during 2010. In addition, genotype 4 increased between 2002 and 2003 (4.0% versus 8.2%, *P* = 0.038 by *χ* ^2^ test) as well as 2010 and 2011 (4.2% versus 10.5%, *P* = 0.0417 by *χ* ^2^ test). Genotype 2 (unsubtypeable), genotype 1 (unsubtypeable), and subtype 1a showed a light and irregularly increased values during the eleven years considered. In order to better address our findings, we compared the distribution of HCV genotypes, considering data among different age groups in 1,217 patients (672 males/545 females) observed during the period 2001–2005 and in 936 patients (476 males/460 females) evaluated during the period 2006–2011. Between the two periods, gender distribution showed differences with a substantial higher number of male only in the first one. Moreover, the two periods considered allowed us to more accurately analyze the possible dynamics in the spread of the different HCV genotypes, as reported by other investigators [9–11], who split in two or more periods of time their HCV patients.

When data of the two periods were examined, we observed within the first four age groups (<25–55) a similar trend for total genotypes within a class age, while HCV rate in older patients showed substantial changes. In particular, a higher percentage of HCV infection in >65 age group (33.7% versus 41.2%) was observed (Table 3). The distribution of HCV genotypes changed according to age in both periods. Patients infected with subtype 1b and 2a/2c were older than those infected by genotype 3 and subtypes 1a and 1a/1b (36–45 age group). Between 2001 and 2005, the distribution of subtype 1b was similar in 56–65 and >65 age groups, while between 2006 and 2011 data showed an increased distribution in >65 age group with respect to 56–65 age group. HCV subtype 2a/2c was prevalent in over 65 patients and for the 2006–2011 period a further increase in this age group was observed. The rate of genotype 3, the most common type in 36–45 age group and in 26–35 age group did not change in both time periods examined, while the number of under 25 years old infected patients decreased between 2006 and 2011. Interestingly the peak of genotype 4 infected cases observed in patients 56–65 years old in the period 2001–2005 split apart into two different peaks during the 2006–2011 period: 27.6% among the 36–45 year old and 24.1% in >65 year old patients (Table 3). 

**Figure 2:**
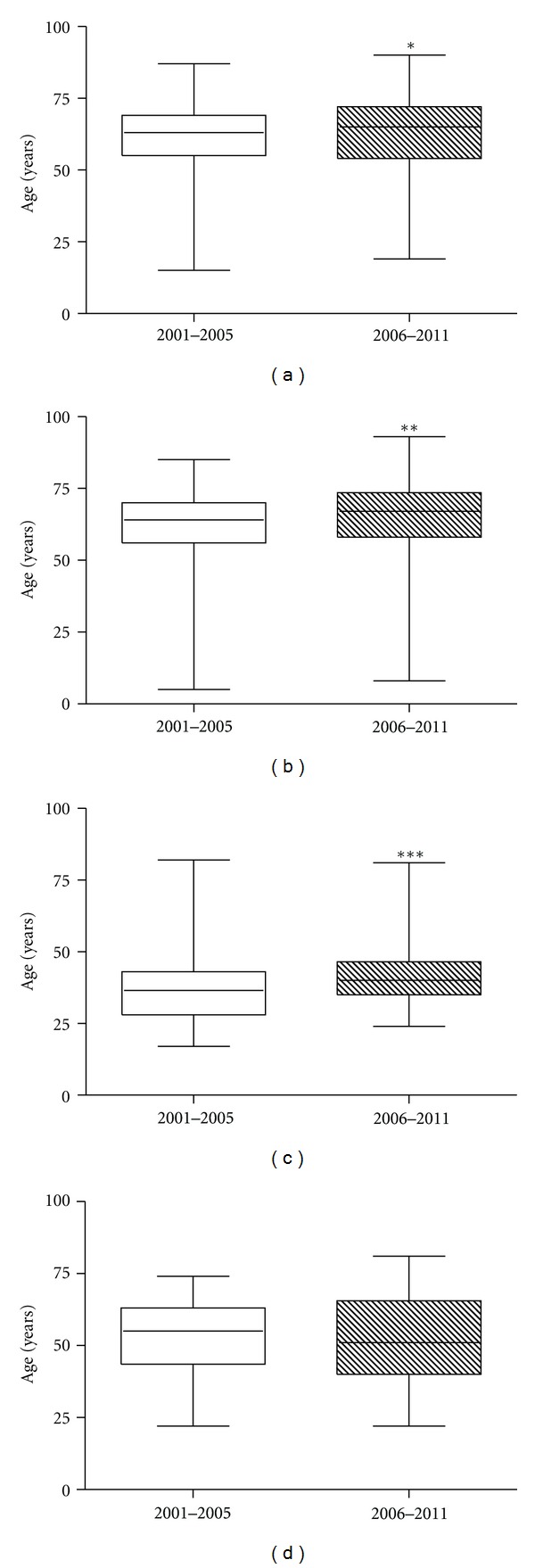
Box plot of HCV genotype 1b (a), genotype 2a/2c (b), genotype 3 (c) and genotype 4 (d) distribution by patient age in the 2001–2005 and 2006–2011 periods of time. *P = 0.0035 versus 2001–2005 by Mann-Whitney test. **P = 0.0083 versus 2001–2005 by Mann-Whitney test. ***P = 0.0009 versus 2001–2005 by Mann-Whitney test.

Statistical evaluation of the age of HCV patients stratified by genotypes, revealed a slight but significant increase by Mann-Whitney test in the median age of 1b (*P* = 0.0035), 2a/2c (*P* = 0.0083), and 3 (*P* = 0.0009) HCV genotype-infected subjects during the 2006–2011 period, whilst genotype 4 patients exhibited a decrease in the median age during the same period studied (Figure 2). 

## 4. Discussion 

In the present study, the main demographic characteristics, as well as the genotype distribution and its dynamic in 2,153 new HCV RNA positive patients living in Calabria, Southern Italy, over an 11-year period were determined. 

The epidemic of HCV infection in Europe is often changing and epidemiological parameters such as prevalence and genotype distribution underwent variations during last decade. Eradication of transfusion-associated infections and improvement in health-care-related standards affected some genotype spreads, which are showing a decreasing trend [12]. On the other hand, permanent presence of intravenous drug users (IDU) [13, 14], broader unsafe sexual practice and a sharp enhancement in immigration from endemic areas [9] could contribute to increase the prevalence of unusual genotypes and/or might account for the introduction of some types that have never been observed before. 

The distribution of HCV genotypes identified in the present study is almost similar to that previously observed [6], with the predominance of subtype 1b, followed by subtypes 2a/2c and genotype 3, however some interesting findings have to be addressed. Traditionally, and still today, in our region subtype 1b represents the most common HCV type, found mostly in elderly patients (over 65) and associated with risk factors as blood/blood derivatives transfusion and surgical procedures. Our data show that its prevalence decreased over the past eleven years and this finding, according to European reports, is mainly due to the improved health standards which reduced its transmission [15]. 

While subtype 2a/2c (the second most prevalent), as well as genotype 3, did not vary significantly with time, interestingly genotype 4 is to date the fourth prevalent genotype in our area. In a previous study, the percentages of HCV genotype 4 infections, related to 1,517 patients observed in a timespan of five years (January 1997–February 2001), exhibited a substantial increase [6], up to 4.7% rate reported during January 2000–February 2001 period. 

Despite HCV-1b rate decrease, our data underlined that, the further increase of genotype 4 up to 6.2%, in the whole period studied may sound as a challenge for epidemiologists, infectious disease specialists, and microbiologists. On the other hand, increasing rate of HCV-4 was not continuous for all the eleven years studied; however, a statistically significant increase was observed between 2002 and 2003, as well as during the 2010 and 2011 period.

A recent study on 3,577 patients from 19 different regions showed that Italy is the European country with the highest prevalence of HCV, with the subtype 1b as the prevailing, and equally distributed all over the Italian area, followed by subtype 2c, genotype 3 and 4 [4]. The prevalence data and age distribution indicated two HCV infection transmission patterns; the first, related to subtype 1b and 2c, mostly found in adults over 60 and reportedly due to the past unsafe health practices, while the second, characteristic of younger people, associated with genotype 3 and 4 and due to drug use and immigration [4].

Since our study was carried out on a resident Italian population, the observed HCV-4 increase could be related to the spread by sexual or intravenous exposure. HCV-4 has been reported to be associated to IDU infection, the more frequent transmission pattern in Western countries [16]; consistently a substantial percentage (data not shown) of our HCV-4 infected patients was IDU subjects.

If we consider the overall observation time, our data show that the middle-age group showed a higher rate of infection by genotype 4 than younger or older group, but when we consider the two periods of our study, we noticed that the highest prevalence of genotype 4 has shifted towards younger age group (36–45) during 2006–2011. Probably this trend could be related to sexual risk behaviour. Even if such shift in age distribution could be in part explained by migrants, mostly illegal, arrived from Middle East and North Africa since two decades ago that contributed to HCV-4 circulation in the patients reported in this study. Moreover travelling for business or leisure to HCV-4 endemic areas has been increasing during the last 20 years with many people from Western countries (including Italy and Calabria) visiting North Africa. 

Genotype 5, mainly reported in South Africa, is sporadically present in different European countries in older patients as we observed. The source of infection is unknown but parenteral iatrogenic routes of transmission have contributed to infection [17]. 

Recently a changed epidemiology profile of hepatitis C virus infection in Europe, as well as in Italy has been observed; HCV genotype shift seems to be mainly due to the modified risk factors and different genotype susceptibility to antiviral drugs [18]. The trend observed since the first epidemiological investigation, carried out in Calabria [4] until the data discussed in the present paper, mostly shows an overall prevalence increase of HCV genotype 4. 

The peculiar diagnostic/clinical/therapeutic features of HCV-4, its significantly increasing rate during 2002-2003 and 2010-2011 periods and the decrease in the median age of HCV-4 patients during 2006–2011 are major concerns, which warrant a deeper investigation to better control such genotype.

## Figures and Tables

**Table 1:** Distribution of HCV genotypes evaluated in 2,153 patients from 2001 to 2011.

	Number of patients (%)										
Genotypes/subtypes	2001	2002	2003	2004	2005	2006	2007	2008	2009	2010	2011
1b	163 (52.1)	150 (59.5)	112 (57.1)	114 (48.5)	108 (48.8)	83 (43.7)	62 (44.7)	63 (40.9)	82 (50.0)	62 (42.5)	60 (42.0)
2a/2c	76 (24.3)	42 (16.7)	36 (18.4)	59 (25.1)	45 (20.4)	56 (29.5)	37 (26.6)	31 (20.1)	31 (18.9)	34 (23.3)	35 (24.5)
3	16 (5.1)	17 (6.7)	9 (4.6)	17 (7.2)	19 (8.6)	12 (6.3)	11 (7.9)	14 (9.1)	13 (7.9)	18 (12.3)	14 (9.8)
4	22 (7.0)	10 (4.0)	16 (8.2)	13 (5.6)	15 (6.8)	13 (6.8)	5 (3.6)	8 (5.2)	11 (6.7)	6 (4.2)	15 (10.5)
1a	9 (2.9)	5 (2.0)	2 (1.0)	4 (1.7)	9 (4.1)	3 (1.6)	6 (4.3)	13 (8.5)	18 (10.9)	14 (9.6)	7 (4.9)
1^∗^		2 (0.8)	5 (2.6)	19 (8.0)	15 (6.8)	13 (6.8)	6 (4.3)	10 (6.5)	3 (1.8)	9 (6.2)	8 (5.5)
1a/1b	23 (7.3)	18 (7.1)	15 (7.6)	3 (1.3)	3 (1.4)	2 (1.1)	2 (1.4)	1 (0.6)	2 (1.3)	1 (0.6)	
2^∗^	3 (1.0)	8 (3.2)	1 (0.5)	5 (2.2)	7 (3.1)	8 (4.2)	10 (7.2)	14 (9.1)	4 (2.5)	2 (1.3)	3 (2.1)
5	1 (0.3)			1 (0.4)							1 (0.7)

Total	313	252	196	235	221	190	139	154	164	146	143

*Unsubtypeable.

**Table 2:** HCV genotype/subtype distribution in the studied population (*n* = 2,153), stratified by gender.

HCV genotypes/subtypes	No. of isolates	Percentage (%)	Gender			
			Male	(%)	Female	(%)
1b	1059	49.2	525	49.6	534	50.4
2a/2c	482	22.4	232	48.1	250	51.9
3	160	7.4	133	83.1	27	16.9
4	134	6.2	78	58.2	56	41.8
1a	90	4.2	58	64.4	32	35.6
1^∗^	90	4.2	49	54.4	41	45.6
1a/1b	70	3.3	44	62.9	26	37.1
2^∗^	65	3.0	27	41.5	38	58.5
5	3	0.1	2	66.7	1	33.3

Total	2153		1148	53.3	1005	46.7

*Unsubtypeable.

**Table 3:** HCV genotype/subtype prevalence among different age groups (years) in total 2,153 HCV RNA-positive patients, stratified in two time periods (2001–2005; 2006–2011).

Number of patients (%)														
	≤25		26–35		36–45		46–55		56–65		>65		Total of age groups	
Genotypes/subtypes	2001/05	2006/11	2001/05	2006/11	2001/05	2006/11	2001/05	2006/11	2001/05	2006/11	2001/05	2006/11	2001/05	2006/11
1b	7 (1.1)	9 (2.2)	34 (5.3)	17 (4.1)	35 (5.4)	36 (8.7)	94 (14.5)	48 (11.7)	234 (36.2)	103 (25.0)	243 (37.5)	199 (48.3)	647	412
2a/2c	6 (2.3)	5 (2.2)	7 (2.7)	12 (5.4)	23 (8.9)	8 (3.6)	25 (9.7)	28 (12.5)	82 (31.8)	46 (20.5)	115 (44.6)	125 (55.8)	258	224
3	13 (16.7)	3 (3.7)	23 (29.5)	21 (25.6)	32 (41.0)	35 (42.7)	8 (10.2)	19 (23.2)	1 (1.3)	2 (2.4)	1 (1.3)	2 (2.4)	78	82
4	1 (1.3)	1 (1.7)	8 (10.5)	3 (5.2)	14 (18.4)	16 (27.6)	18 (23.7)	15 (25.9)	24 (31.6)	9 (15.5)	11 (14.5)	14 (24.1)	76	58
1a	1 (3.4)	3 (4.9)	6 (20.7)	11 (18.0)	12 (41.5)	20 (32.8)	5 (17.2)	17 (27.9)	1 (3.4)	6 (9.8)	4 (13.8)	4 (6.6)	29	61
1^∗^			6 (14.6)	4 (8.2)	11 (26.8)	9 (18.4)	9 (22.0)	6 (12.2)	5 (12.2)	13 (26.5)	10 (24.4)	17 (34.7)	41	49
1a/1b	2 (3.2)		9 (14.5)		23 (37.1)	3 (37.5)	7 (11.3)	3 (37.5)	10 (16.2)	1 (12.5)	11 (17.7)	1 (12.5)	62	8
2^∗^		1 (2.4)			1 (4.2)	3 (7.3)	6 (25.0)	6 (14.6)	4 (16.6)	8 (19.5)	13 (54.2)	23 (56.2)	24	41
5											2 (100.0)	1 (100.0)	2	1

Total (%)	30 (2.5)	22 (2.4)	93 (7.6)	68 (7.3)	151 (12.4)	130 (13.9)	172 (14.1)	142 (15.2)	361 (29.7)	188 (20.0)	410 (33.7)	386 (41.2)	1217	936

*Unsubtypeable.
